# Effects of low power laser irradiation on bone healing in animals: a meta-analysis

**DOI:** 10.1186/1749-799X-5-1

**Published:** 2010-01-04

**Authors:** Siamak Bashardoust Tajali, Joy C MacDermid, Pamela Houghton, Ruby Grewal

**Affiliations:** 1 Department of Physical Therapy, Elborn College, The University of Western Ontario, London, Ontario, N6G 1H1, Canada; 2 Hand and Upper Limb Centre Clinical Research Laboratory, St Joseph's Health Centre, 268 Grosvenor St, London, Ontario, N6A 3A8, Canada; 3 Department of Surgery, Hand and Upper Limb Centre, Clinical Research Laboratory, St Joseph's Health Centre, 268 Grosvenor St, London, Ontario, N6A 3A8, Canada

## Abstract

**Purpose:**

The meta-analysis was performed to identify animal research defining the effects of low power laser irradiation on biomechanical indicators of bone regeneration and the impact of dosage.

**Methods:**

We searched five electronic databases (MEDLINE, EMBASE, PubMed, CINAHL, and Cochrane Database of Randomised Clinical Trials) for studies in the area of laser and bone healing published from 1966 to October 2008. Included studies had to investigate fracture healing in any animal model, using any type of low power laser irradiation, and use at least one quantitative biomechanical measures of bone strength. There were 880 abstracts related to the laser irradiation and bone issues (healing, surgery and assessment). Five studies met our inclusion criteria and were critically appraised by two raters independently using a structured tool designed for rating the quality of animal research studies. After full text review, two articles were deemed ineligible for meta-analysis because of the type of injury method and biomechanical variables used, leaving three studies for meta-analysis. Maximum bone tolerance force before the point of fracture during the biomechanical test, 4 weeks after bone deficiency was our main biomechanical bone properties for the Meta analysis.

**Results:**

Studies indicate that low power laser irradiation can enhance biomechanical properties of bone during fracture healing in animal models. Maximum bone tolerance was statistically improved following low level laser irradiation (average random effect size 0.726, 95% CI 0.08 - 1.37, p 0.028). While conclusions are limited by the low number of studies, there is concordance across limited evidence that laser improves the strength of bone tissue during the healing process in animal models.

## Background

Bone and fracture healing is an important homeostatic process that depends on specialized cell activation and bone immobility during injury repair [1,2]. Fracture reduction and fixation are a prerequisite to healing but a variety of additional factors such as age, nutrition, and medical co-morbidities can mediate the healing process [3,4]. Different methods have been investigated in attempts to accelerate the bone-healing process. Most studies have concentrated on drugs, fixation methods or surgical techniques; however, there is a potential role for adjunctive modalities that affect the bone-healing process.

Laser is an acronym for "Light Amplification by stimulated Emission of Radiation" [5]. The first laser was demonstrated in 1960 and since then it has been used for surgery, diagnostics, and therapeutic medical applications [6]. The physiological effects of low level lasers occur at the cellular level [7,8], and can stimulate or inhibit biochemical and physiological proliferation activities by altering intercellular communication [9]. Early work on physical agents as mediators of bone healing was performed by Yasuda, Noguchi and Sata who studied the electrical stimulation effects on bone healing in the mid 1950s [1,10]. In subsequent years, others repeated this work in humans [1,11] and a variety of physical agents have been investigated as potential mediators of bone healing [12-16]. With increasing availability of lasers in the early 1970s, the potential to investigate its use as a modality to affect the healing of different connective tissues became possible [17-19]. In 1971, a short report by Chekurov stated that laser is an effective modality in bone healing acceleration [19].

Subsequently, other researchers studied bone healing after laser irradiation using histological, histochemical, and radiographic measures [18-24]. These studies have demonstrated mixed results where some observed an acceleration of fracture healing [19,21-24], while others reported delayed fracture healing after low-level laser irradiation [20,25].

In 1996, David and his colleagues presented the first biomechanical evaluation of bone healing after laser irradiation [25]. They did not find any positive changes in biomechanical bone properties after laser irradiation, and concluded that low power laser irradiation did not help to promote bone healing. David and his colleagues stated that their results were more valid than previous studies because they used objective biomechanical outcome measures rather than subjective methods such as histology or radiology [25]. A single study has not definitive results because it cannot address different types of fractures, dosages, or mediating factors that might influence the potential role for low-power laser across different constructs. However, this study did define the need for additional biomechanical research to identify the role for low-power laser across different fracture constructs and the need for definitive biomechanical measures of bone strength in such studies.

The purpose of this study was to conduct a systematic review and meta-analysis of animal studies that investigated low-level laser irradiation effects on bone healing. Our inclusion criteria required that studies have a quantitative biomechanical measures of bone strength since this is considered the most reliable and definitive indicator of bone healing in animal studies [25,26].

## Methods

A systematic search of five electronic databases including MEDLINE from 1966 to October 2008; and EMBASE, Pubmed, CINAHL and Cochrane from 1980 to October 2008 was conducted using an iterative strategy. The search was repeated following review of the eligible papers to specifically search for the biomechanical outcome measures identified within the initial retrieval.

The researchers also reviewed the bibliographies of all retrieved articles to identify possible additional studies. One researcher did a hand search of one journal known to publish in the area of interest of study (Osteosynthesis and Trauma Care) from September 2002 to December 2003. Two researchers independently checked the inclusion criteria in the method sections of each eligible article. The inclusion criteria of this systematic search were: 1) live animals subjects; 2) a long bone fracture or deficiency model was created; 3) random allocation of treatment; 4) any type of low level (power) laser irradiation was provided as an intervention to at least one of the treatment groups; 5) a quantitative measure of bone biomechanics was performed; 6) English language. Abstracts were reviewed by at least two raters to determine if they met eligibility criteria.

The most common reasons for excluding articles were lack of data from an animal fracture model and in particular measures of bone biomechanics. Histology, radiology, and histomorphometry measurement methods were the most commonly methods used to monitor bone healing in located articles. Through the abstract review, we excluded articles that clearly referred to a surgical laser device or used laser as an outcome measurement (Laser Doppler). All remaining abstracts were reviewed as the full paper articles. A total of 49 full papers were reviewed as full text to determine eligibility.

**Figure 1:**
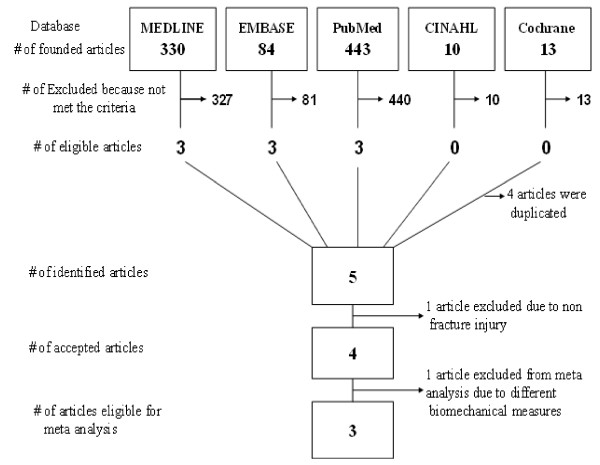
**Flow diagram for identification the eligible experimental control animal studies evaluating the effect of low power laser irradiation on bone healing based on biomechanical bone properties**.

Of the 49 potential relevant papers only five articles met the inclusion criteria and reported on the effects of laser irradiation effect on biomechanical properties of bone during a fracture healing model (Figure 1). One article (Akai et al) [27] that evaluated biomechanical properties of bone was excluded at full text review because it did not include a fracture model and evaluated bone biomechanical properties after joint immobilization. Another article [28] was also excluded from the meta analysis, since the authors (Teng et al) used two different biomechanical bone properties as the outcome measurements (the anti-torsion torque and the torsion-breakage moment). As a result, it was not possible to match and calculate Teng biomechanical results with data from the other articles data in a meta analysis. However, we assessed the quality of Teng article base on the QATRS and common quality measurements methods.

Three articles [25,26,29] were entered into meta analysis, since these three had a common metric biomechanical measures (maximum force), whereas one [28] used another biomechanical measures (the anti-torsion torque and the torsion-breakage moment). A time point where data was retrievable across all three studies was selected for meta analysis. Thus, the maximum bone tolerance force (Maximum force or F-max.) four weeks following fracture was defined as main biomechanical bone properties for the meta analysis. Figure 1 summarizes the search strategy and keywords review [See Additional File 1].

Potentially eligible articles were printed, reviewed and critically appraised for quality rating by two independent reviewers. Systematic reviews are commonly performed in human research but rarely in animal research. Quality rating scales commonly used in human research may not be appropriate for the animal studies, since they do not consider issues like the appropriateness of the animal model to construct being evaluated. The second author (JM) developed a quality rating scale for animal/tissue research scale (QATRS) questionnaire to assess the quality of animal studies. The QATRS is a 20-point scale evaluation chart that is designed based on randomization, blinding, similarity of animal/tissue model with human application, standardization and reliability of measurement techniques, the management of study withdrawals, and appropriateness of statistical methods [See Additional File 2].

Two raters reviewed all four papers using the structured critical appraisal tool designed for studies evaluating interventions in animal models independently (QATRS). We arbitrarily classified the quality of the animal studies by defining cut off scores for quality as excellent, moderate, low and very low quality based on their overall score on the scale (16-20, 11-15, 6-10, 5 or lesser, respectively). We also performed a similar critical appraisal using Jadad* and PEDro** methods [See Additional File 3], to find how much our quality animal research scale is close with the common quality studies measurement method (Table 1). The Jadad and PEDro quality measurement methods are used for human studies [30,31], and were not altered to apply specifically for the animal studies. We use these previously published scales to cross validate our quality measurement (QATRS) scores. There was complete agreement between the reviewers on the score of eligible articles.

**Table 1:** Maximum force (Mean + SD), Effect Sizes and Quality Score of Included Studies

Mean maximum force (SD)									
		Sample size		4 weeks after fracture			Quality score		
Trial	Location of fracture	Treatment group	Control group	Treatment group	Control group	Effect Size	PEDro/10	Jadad/5	QATRS/20
David et al	Tibia (Mid portion)	62	62	a) 1630 (1020)	1340 (540) (1)	0.36	5	0	12
				a) 1120 (900)	1190 (570) (2)	-0.09			
				b) 1110 (650)	1510 (820) (1)	-0.30			
				b) 670 (680)	1020 (890) (2)	-0.40			

Luger et al	Tibia (Mid portion)	25	25	74.4 (43.1)	46.5 (20.2) (1)	0.82	7	3	17

Tajali et al	Tibia (4 cm below tibial tubercle)	30	30	36.82 (7.42)	27.79 (6.14) (2)	1.34	7	1	15

Teng et al	Radius	8	16*	N/A	N/A	--	6	2	13

* 8 samples for He-Ne and 8 samples for Co2, (1) F Plan: Vertical (Sagital) Plan, (2) T Plan: Horizontal Plan, a) 2 (J) laser irradiation per session, b) 4 (J) laser irradiation per session

### Data Extraction

Two researchers independently extracted the data from each eligible article. All authors evaluated bone-healing process based on biomechanical bone properties as the objective index assessment, but the biomechanical variables were different between the studies. The researchers coded all related variables. The coded variables were: a) animal type, b) animal race, c) sex, d) age, e) weight, f) evaluation surface, g) evaluation time (week), h) type of surgery, i) type of fixation, j) bone type, k) mechanical test, l) speed of test, m) graph type, n) type of laser (independent variable), o) laser output, p) irradiation distance, q) irradiation time per day, r) number of treatment sessions, s) irradiated energy per day, t) total irradiated energy, u) dependent variables (including: maximum force, callus area, stress high yield, extension maximum load, callus stiffness, energy absorbed capacity, deformation, ultimate bending strength, force at elastic stage, anti-torsion torque, torsion-breakage moment) (Table 2).

**Table 2:** The Biomechanical Bone Properties (Dependent Variables) of Included Studies.

Authors	Biomechanical Bone Properties (Dependent Variables)
David et al., 1996	Force - Deflections Values

Luger et al., 1998	Maximum load, Callus area, Stress high yield, Extension Maximum, Callus stiffness

Tajali et al., 2003	F - Max, Energy absorbed capacity, Deformation, Ultimate bending strength, Force at elastic stage

Teng et al., 2006	Anti - torsion torque, Torsion - breakage moment

### Statistical Analysis

The Q statistic was calculated to test the homogeneity of studies. A significant Q statistic indicates the presence of between study variance that is not consistent with study sampling error [32]. A significant p value in homogeneity test would indicate that the studies are heterogeneous and are not measuring an effect of the same size [33]. On the contrary, if the studies are not heterogeneous, the studies results are considered similar and therefore they can be combined [34] (Table 3).

**Table 3:** Computed Random effect size, CI95 and Q value (Heterogeneity test).

**Model**	**Effect size and 95% confidence interval**				**Test of null (2-Tail)**			**Heterogeneity**	
				
Model	Number Studies	Point estimate	Lower Limit	Upper Limit	Z-value	P-value	Q-value	df (Q)	P-value
Random	3	0.726	0.079	1.373	2.199	0.028	2.652	2	0.196

There are two types of statistical models, which can be used for effect size calculation in meta analysis; fixed effects model and random effects model [32]. The homogeneity of effect sizes has been associated with the selection of fixed versus a random effects method of analysis [32]. Both random and fixed effects models are used to determine the statistical differences of the combined results; however, the random effects model is advised when there is an evidence of heterogeneity in variance (Hedges & Vevea, 1998) [32]. We chose the random effects model because the random model is more conservative [33] and it is also advised when the authors want to generalize their findings [32]. Effect sizes for the studies were calculated by using the equation [35].

Where d is the effect size; *mt *is the mean change of maximum force in the treatment group; *mc *is the mean change of maximum force in the control group; and *s *is the pooled SD between *mt *and *mc*. We used this equation to calculate the pooled SD [36].

Where *nt *and *nc *are the sample size of the treatment and control groups; and *S *^*t *^and *S*^*c *^are the standard deviations of the treatment and control groups. The effect sizes were reported as standardized mean differences and 95% CI and the fixed effects model were run to determine the statistical differences of the results. The effect size (d) values of 0.20, 0.50, and 0.80 were considered as the small, medium, and large effect sizes, suggested by Cohen authors [32]. All data were entered into Comprehensive Meta Analysis (CMA) program [37] to provide a Z value and to construct the forest plots to show the overall effect size and the related %95 CI.

We also evaluated the bias of publication via analysis option by Fail Safe N computation in CMA. The Fail Safe N can be calculated by the equation K_0 _= K (Mean d - d_trivial_)/d_trivial_, where K_0 _is the number of needed studies to produce a trivial effect size, K is the number of studies in meta analysis, Mean d is the mean effect size from all studies, d_trivial _is the estimate of a trivial effect size [32].

Finally, we evaluated to what extent the number of treatment sessions can be considered a moderator variable. Therefore, we stratified the articles data based on the number of treatment sessions and then compared them by t test and ANOVA measurement methods through CMA [37].

## Results

### Description of studies

Descriptive information of all eligible studies is shown in Tables 4, 5 and 6. Among three selected studies for the final analysis, two studies (Luger et al., and Tajali et al.) supported the positive effects of low-level laser irradiation on bone healing and one researcher (David et al.) did not find a significant effect for laser effectiveness on bone healing. Two studies (Luger et al. and Tajali et al.) evaluated the bone healing process using only biomechanical measurements, while another (David et al) also used histology and radiology measurement methods.

**Table 4:** Study Characteristics of Selected Experimental Controlled Animal Studies on He-Ne Low Level Laser Irradiation Effects on Bone Healing

Authors	Animal Type	Animal Race	Gender	Age	Weight (gr)	Evaluation Surface	Evaluation Time (Week)
David et al., 1996	Rat	Sprague - Dawely	Female	N/A	225 -300	Horizontal (T) & Vertical (F)	2 - 4 - 6

Luger et al., 1998	Rat	Wister	Male	4 month	400 ± 20	Vertical (Sagital)	4

Tajali et al., 2003	Rabbit	Dutch	Male	4-6 Month	1600-2000	Horizontal	2 - 3 - 4

Teng et al., 2006	Rabbit	New Zealand	Male	N/A	2000-2500	N/A	35 (Days)

**Table 5:** Study Characteristics of Selected Experimental Controlled Animal Studies on He-Ne Low Level Laser Irradiation Effects on Bone Healing

Authors	Surgery Type	Type of Fixation	Bone Name	Mechanical Test	Test Speed (mm/min)	Graph Type	* Laser Type
David et al., 1996	CO	IF (Intramedullary 1/32" Kirschner wire)	Tibia	Four Point Bending Test	5	Stress-Strain	He - Ne

Luger et al., 1998	CO	IF (Kirschner wire)	Tibia	Tension - Stress Test	5	Load-Strain	He - Ne

Tajali et al., 2003	PO	EF	Tibia	Three Point Bending Test	N/A	Load-Deformation	He - Ne

Teng et al., 2006	PO	Without Fixation	Radius	Biomechanics Anti - Torsion Test	N/A	N/A	He - Ne & Co2

CO = Complete Osteotomy, PO = Partial Oasteotomy, IF = Internal Fixation, EF = External Fixation,

* Independent Variable

**Table 6:** Study Characteristics of Selected Experimental Controlled Anima Studies on He-Ne Low Level Laser Irradiation Effects on Bone Healing

Authors	Laser Output (mw)	Distance between Producer and Skin (cm)	Irradiation Time per Day (min)	Number of treatment sessions	Irradiated energy per session	Total Irradiated Energy		
David et al., 1996	10	N/A	N/A			2 week	4 week	6 week
						
				(2 week) 6	0	0	0	0
				(4 week) 13	2	12	26	40
				(6 week) 20	4 (J) every other day	24	52 (Joules)	80

Luger et al., 1998	35	20	** 30	14	***	***		
					21 J (each area)	294 (J) (each area)		
					63 J (in total)	882 (J) (in total)		

Tajali et al., 2003	2	N/A	** 30	14	1.2 (J/cm2)	16.8 (J/cm2) ***		
				21		5.2 (J/cm2)		
				28		33.6 (J/cm2)		

Teng et al., 2006	N/A	N/A	10	35	***	***		
					He-Ne: 16.8 (J/cm2)	He - Ne: 588 (J/cm2)		
					Co2: 90 (J/cm2)	Co2: 3150 (J/cm2)		

** Including 10 minutes on fracture area, 10 minutes on the area above the point of fracture, and 10 minutes on the area below the fracture. *** Meta analysis authors calculated amount of irradiated energy based on the articles data with this equation [43]:

Set Power (w) * Time (s) = Total Amount of Energy (J)

Total Amount of Energy/Treatment Surface (cm2) = Energy Density (J/cm2)

All studies measured the biomechanical bone healing changes four weeks after fracture. David measured the bone healing changes 2, 4 and 6 weeks after fracture, Luger checked these measurements just 4 weeks after the fracture, and Tajali did the biomechanical measurements 2, 3 and 4 weeks after bone deficiencies (Table 7). Two authors (Luger et al and Tajali et al) applied intervention to separate experiment and control groups, while the other author (David et al) operated both hind limbs of the animals and considered one limb as the experiment and the other limb as the control. This approach may be questionable, as it could not control the systematic effects of low power lasers irradiation [38-40].

**Table 7:** Maximum force (Mean + SD) 2, 3, 4 or 6 weeks after fracture or surgery.

Authors	2 week	3 week	4 week	6 week
			2 Joules/day	
			
David et al. (1996)	N/A	N/A	E 1630 ± 1020 *	E 1880 ± 1080 *
			C 1340 ± 540 *	C 2330 ± 1210 *
				
	N/A	N/A	E 1120 ± 900 **	E 1750 ± 1060 **
			C 1190 ± 570 **	C 2330 ± 1050 **
				
			4 Joules/day	
			
	N/A	N/A	E 1110 ± 650 *	E 2480 ± 1140*
			C 1510 ± 820 *	C 2000 ± 680 *
				
	N/A	N/A	E 670 ± 680 **	E 1680 ± 1280 **
			C 1020 ± 890**	C 2280 ± 140 **

Luger et al. (1998)	N/A	N/A	E 74.4 ± 43.1*	N/A
			C 46.5 ± 20.2*	

Tajali et al. (2003)	E 28.82 ± 8.19**	E 29.85 ± 5.50**	E 36.82 ± 7.42**	N/A
	C 24.44 ± 3.19**	C 27.70 ± 5.32**	C 27.79 ± 6.14**	

Teng et al. (2006)	NA	NA	NA	NA

E = Experiment, C = Control; * Data refers to biomechanical evaluation in vertical plan; **Data refers to biomechanical evaluation in horizontal plan.

Fixation also varied across the studies; internal fixation (k-wires) was used in two studies (David et al. and Luger et al.), while external fixation was preferred in the other article (Tajali et al.). All three eligible studies used the low power He-Ne laser as their independent variable.

Laser treatment parameters varied markedly across studies. All three studies included a treatment of He-Ne laser at a wavelength of 632.8 nm, which would have resulted in similar absorption properties in the target area. However, none of the studies provided complete descriptions of laser dosage, treatment parameters and application techniques. Therefore, it was not possible to compare the amount of laser energy delivered in the included studies. David et al (1996) reported the amount total irradiated energy, but did not explain the irradiation application technique. In the study performed by Tajali et al (2003), a grid technique was used to apply laser irradiation to each square centimeter of tissue; however the number of points over which laser was applied was not defined. Luger et al (1998) used and applied the laser at a distance of 20 cm from the skin, which would have significantly reduced total energy delivered to the target tissue. All studies evaluated biomechanical properties of the bone at 4 weeks post fracture. David used the laser irradiation every other day during the period of study, and Luger and Tajali used laser irradiation on a daily basis. Luger stopped treatment after 14 days whereas the other studies continued daily treatments for at least 4 weeks (Tables 4, 5, 6).

#### Outcomes measured

The eligible studies used different indicators of the biomechanical properties indicating bone healing. There were 11 biomechanical bone properties measured. Maximum bone force tolerance (Maximum Force) was considered the major dependent variables in three studies (out of four). The other biomechanical variables were different from study to study. Although David et al (1996) studied just one main biomechanical variable (Maximum Force), they also used histological and radiological assessment methods. Luger et al (1998) studied callus area, stress high yield, extension maximum load, and callus stiffness as the biomechanical variables. Tajali et al (2003) studied energy absorbed capacity (EAC), deformation, ultimate bending strength (UBS), and force at elastic stage as the biomechanical variables (Table 2).

#### Calculation of effect size

The maximum bone tolerance force before the point of fracture was the most common biomechanical variable in all eligible studies and was used to calculate effect size of each article in this meta analysis. A total of 234 samples across all three identified studies were entered in the meta analysis based on the maximum force. We chose to evaluate the biomechanical data 4 weeks following surgery or fracture. We chose this as a clinically relevant endpoint, since earlier time may not have demonstrated sufficient healing [25,26,29], and also expect that healing would be completed in both the experiment and control groups at later time points [26,29]. Although the time points for biomechanical evaluation was different in each study (Table 4), all eligible articles performed a biomechanical evaluation at 4 weeks after surgery or fracture allowing us to perform data synthesis on a common metric.

David et al. [25] measured the force maximum variable changes with two different doses of low power He-Ne laser irradiation (2 and 4 Joules per/day), while the other researchers (Luger and Tajali) used one dosage for all experiment groups (Table 6). To standardize the doses used in each study, we calculated an average effect size between two effect sizes of force maximum changes in David article by CMA program. All effect sizes were calculated by SPSS and CMA [37].

#### Testing for homogeneity of variance

The Q statistic result showed that the value of Q for the samples in this study (n = 3) was not statistically significant (Q 2.652, p 0.196). Therefore, the distribution of the effect sizes was homogenous and we could combine study results. The average effect size demonstrated a statistically significant effect for laser being beneficial in terms of bone strength (n 3, d = 0.73, CI_95 _.08 - 1.38) (Table 3).

#### Merits of different published studies (variables)

The effect sizes of eligible studies were computed by CMA to evaluate the merits of different published studies (Table 1). The CI_95 _for maximum force F-max includes zero, indicating there is no significant difference in terms of force maximum in the study by David et al (1996) (mean 0.072, 95% CI - 0.976 - 1.120, p 0.89). The effect size in David article [25] was not statistically significant. The average effect size in David article for two different dosage (2 and 4 J/day) 4 week after surgery is equal d = - 0.072 which shows the low effect size in this article. On the contrary, the CI_95 _for F-max in Luger study (mean 0.820, 95% CI 0.087 - 1.553, p 0.028), and also the CI_95 _for F-max in Tajali study (mean 1.400, 95% CI .137 - 2.662, p 0.030) showed high effect sizes in these two articles and the statistical significant differences.

Calculation of pooled standard deviation and average effect size in each article showed the lowest effect size for David study [25]. This study also had relatively low quality scores (QATRS 12/20, Jadad 0/5, PEDro 5/10). On the contrary, Luger and Tajali studies [26,29] had larger effect sizes (more than high limit of effect size for good articles d > 0.80). The quality evaluation results of these articles also showed good quality for Luger and Tajali (QATRS 17/20, Jadad 3/5, PEDro 7/10 for Luger et al article, and QATRS 15/20, Jadad 1/5, PEDro 7/10 for Tajali et al article).

**Figure 2:**
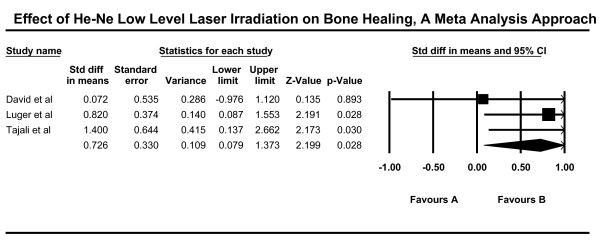
**The forest plot of the random effects model based on bone biomechanical properties (force maximum) changes 4 weeks after bone injury**.

In summary, the average effect size calculation of force maximum, 4 week after bone injury in eligible articles shows that one article has low value effect size (David et al d = 0.072), and two articles have excellent value effect size (Luger et al d = 0.82, Tajali et al d = 1.400). The computed random effect size (mean 0.726, 95% CI 0.079 - 1.373, p 0.028) suggests main research hypothesis that low power laser irradiation can increase bone-healing process in animal samples based on an evaluation of biomechanical bone properties (Figure 2).

#### Fail Safe N and the number of treatment sessions

The results of Fail Safe N calculation showed that 38.28 (= 39) more unpublished articles are needed to nullify our results. The d results also showed that it is possible to divide the number of treatment sessions to three parts: a) Less than 14 Treatment sessions, b) Between 14 to 21 Treatment sessions, and c) 28 Treatment sessions. There was no significant difference between experimental and control groups after 14 treatment sessions (mean - 0.072, 95% CI - 1.204 - 1.060, ns). On the contrary, low power laser irradiation for 14 to 21 sessions significantly improved the bone-healing process in animal (mean 0.557, 95% CI 0.079 - 1.035, p 0.022). Finally, 28-session low level laser irradiation caused the significant increase on bone healing process in animal (mean 1.400, 95% CI 0.137 - 2.662, p 0.030) (Table 6, Figure 2).

## Discussion

Three of the four selected articles reported a positive effect of low-level laser therapy on bone healing [26,28,29], and one article reported negative results [25]. Meta analysis revealed that overall positive impact of laser on bone healing. Although there are different kinds of low power lasers e.g. Co2, He-Ne, Ga-Al-As, and Infra Red, all the identified studies used continuous wave He-Ne lasers. This may be because He-Ne laser has some support in earlier studies on connective tissue healing [18,19,22-24]. Teng et al (2006) was the only author who compared the He-Ne with Co2 lasers irradiation effects based on the bone biomechanical properties and also radiology [28]. He reported the composition and biomechanical properties were improved over controls following irradiation for 35 days with either type of laser. However, these results were excluded from the final meta analysis due to non-similarity of biomechanical variables. Nevertheless, it is important to note that the conclusions were in agreement with the present study. Incomplete and inconsistent information provided about laser treatment protocols prevented an evaluation of laser dosimetry. Future studies that compare different wavelengths and amount of laser irradiation are needed to define the optimum application strategy. However, these studies must provide complete information about the power, time (per point applied and the number of points), and area of treatment (beam spot size), so that energy density and total energy delivered with each treatment can be calculated. In this way useful comparisons can be made between studies with regards to laser dosimetry. Although randomization and the use of internal controls can increase power in studies where the effects are localized, the use of two hind limbs of each animal, one as the experiment and the other as the control, in the study by David [25] might lead to a false negative findings, since low level laser therapy has some systematic effects [38-40]. Moreover, surgery or fracture of both hind limbs in each animal, created excessive limitations in normal mobility for animals in David study [25] and may have affected the bone healing process [3]. Finally, the use of intermedullary nails in some experimental groups may affect the study results [41,42], especially when the authors had to remove the nails before the biomechanical assessment and reaming of fractures [41,42] possibly explaining David's negative results. Our meta-analysis was only able to identify a limited number of studies that have addressed the impact of laser on the strength of healed bone in an animal fracture model. Despite these limitations, there was a statistically significant impact of laser on the biomechanical properties of healed bone-particularly in more than 14 sessions laser application. Furthermore, our failsafe n calculation indicates that a large number of contrary studies would be required to refute this finding. This would suggest that sufficient animal research is available to support experimental use of laser for bone healing in humans.

Findings of improved bone healing in animal models with adjunctive laser therapy are consistent with other research on the effects of laser. The cellular reactions such as ATP synthesis promotion, electron transport chain stimulation, and cellular pH reduction might form the basis for the clinical benefits of low-level laser therapy [43,44], and these biochemical and cell membrane changes may increase activities of macrophage, fibroblast, lymphocyte and the other healing cells [45,46]. Increase of collagen and DNA synthesis, faster removal of necrotic tissue [20], increase of Ca deposition [19,21,22], increase of periosteum cells function [18], increase of osetoblast and osteocyte function [18,19], new vascularistion [21,22], stimulation of enchondral ossification, earlier differentiation of mesenchymal cells, increase of preosteogenic cells [23], and stimulation of callus formation [21,22] are some of the positive effects of low level laser therapy on bone healing process which have been reported by former researchers and can explain the bone healing stimulation under low level laser therapy.

### Study Limitations

Our study findings must be viewed with caution at this time because of substantial limitations. 1) It is possible that we missed some published or unpublished related articles. 2) Although the results of random and fix effects models are in favor of laser effects on bone healing (fixed effects model, n3, mean 0.727, CI_95 _0.184 - 1.269, p 0.01), the small sample size of selected studies may cause the insignificance result in Q statistic. 3) We tried to identify a core outcome measure that would allow comparability across studies. Although we ran analysis to check for appropriateness of combining data from analysis, our results were based on the fractures from two different animal types (tibia in rat and rabbit models) [33]. 4) Given the small number of studies we could not formally incorporate quality measurement scores into our synthesis. The results of quality measurement methods and power of the selected studies could not be used in our Meta analysis. 5) The samples in one study (David) were used as the experimental and control at the same time. The data came from this study could not be considered as independent data, but they were still independent from the other eligible studies' data. 6) Although we know that the process of fracture healing is consistent [47], variations in tissue type and depth may have affected the impact of laser. And finally 7) the actual dosage delivered is questionable across the studies given that laser transducer calibration was not mentioned.

## Conclusion

Our meta-analysis identifies that low level laser therapy improves the biomechanical properties of bone following fracture healing in animal models. There is still insufficient evidence to establish optimal dosage, but low-level laser irradiation for at least 14 to 21 sessions was required for preferential effects. The results appear to be sufficient animal evidence of improved bone healing in animal models to warrant clinical trials evaluating the role of low-level laser irradiation on human bone healing.

## Competing interests

The authors declare that they have no competing interests.

## Authors' contributions

SBT carried out the literature search and review, data extraction, synthesized results, prepared the initial draft, performed the statistical analysis, coordinated revisions, submitted the manuscript, and prepared the written draft. JMD contributed to the literature search and review, developed the critical appraisal tool, coordinated the appraisal, and contributed to data critical appraisal and manuscript revisions. PH and RG contributed to the search strategy and revisions of the manuscript. All authors read and approved the final article.

## Supplementary Material

Additional file 1 **The authors selected initial key words from related articles.** Mesh and SCOPUS international data lines were used to find more related key words with close meanings. Click here for file

Additional file 2 The Quality of Animal/Tissue Research Scale. Click here for file

Additional file 3 Jadad and PEDro Quality Measurement methods. Click here for file
