# Supplementary material for: Effects of low power laser irradiation on bone healing in animals: a meta-analysis
Source: J Orthop Surg Res. 2010 Jan 4;5:1. doi: 10.1186/1749-799X-5-1 (PMC2829511; doi:10.1186/1749-799X-5-1)
Supplement: Additional file 1 — The authors selected initial key words from related articles. Mesh and SCOPUS international data lines were used to find more related key words with close meanings. [file 1749-799X-5-1-S1.DOC]

**Additional File 1**

**Title:** Key Words in Search Strategy.

**Description:** The authors selected initial key words from related articles. Mesh and SCOPUS international data lines were used to find more related key words with close meanings;

"Fracture" or "Fractures" or "Fracture healing" or "Fracture healings" or "Bone healing" or "Bone regeneration" or "Fracture regeneration" or "Bone remodeling" or "Fracture remodeling" or "Bone consolidation" or "Fracture consolidation" or "Fracture repair" or "Bone repair" or "Osteosynthesis" or "Osteogenesis" or "Osseointegration" or "Osteoconduction" AND

"Biomechanics" or "Biomechanical properties" or "Bending strength" or "Tensile strength" or "Energy absorbed capacity" or "Deformation" or "Callus stiffness" or "Maximum force" or "Compressive strength" or "Elasticity" or "Friction" or "Shear strength" or "Mechanical stress" or "Torsion" or "Elastic resistance" or "Dissipation of energy" or "Breaking strength" AND

"Laser" or "Lasers" or "Laser therapy" or "Low level laser" or "Low power laser" or "Photo therapy" or "Light therapy" or "Photon" or "Therapeutic light" or "Therapeutic photon" or "Laser biostimulation" or "Photon biostimulation"
