# Supplementary material for: Effects of low power laser irradiation on bone healing in animals: a meta-analysis
Source: J Orthop Surg Res. 2010 Jan 4;5:1. doi: 10.1186/1749-799X-5-1 (PMC2829511; doi:10.1186/1749-799X-5-1)
Supplement: Additional file 2 — The Quality of Animal/Tissue Research Scale. [file 1749-799X-5-1-S2.DOC]

# Additional File 2

**Title:** The Quality of Animal/Tissue Research Scale.

**Description:**

| **Item** | **Rating** | | |
| --- | --- | --- | --- |
|  | Yes (2) | Partial (1) | No  (0) |
| 1. Animals /tissue samples were randomly allocated to groups. |  |  |  |
| 2. The animals/tissue samples were similar across comparison groups. |  |  |  |
| 3. The tissue/animal model study was appropriate for the biological properties/questions being evaluated. |  |  |  |
| 4. The animal model used was appropriate to make inferences in terms of human application? (Tissue similar to, or is human tissue). |  |  |  |
| 5. Objective measurements were performed using sufficient standardization of measurement techniques and appropriate instrumentation. |  |  |  |
| 6. Reliability of measurements was reported or referenced to indicate sufficient consistency of the outcomes analyzed. |  |  |  |
| 7. All animals entered into the study accounted for? (All were analyzed or reasons for withdrawal noted). |  |  |  |
| 8. 90% of the animals entered were included in the data analysis. |  |  |  |
| 9. The between group/time statistical comparisons used appropriate statistical methods. |  |  |  |
| 10. Measures of variability and confidence intervals were provided to indicate the range/size of the effects observed. |  |  |  |
| Total score (/20) |  | | |

© MacDermid 2008
