# Supplementary material for: Effects of low power laser irradiation on bone healing in animals: a meta-analysis
Source: J Orthop Surg Res. 2010 Jan 4;5:1. doi: 10.1186/1749-799X-5-1 (PMC2829511; doi:10.1186/1749-799X-5-1)
Supplement: Additional file 3 — Jadad and PEDro Quality Measurement methods. [file 1749-799X-5-1-S3.DOC]

### Additional File 3

**Title:** Jadad and PEDro Quality Measurement methods.

**Description:** *The Jadad scale is a three-item questionnaire that scores studies from 0 to 5 based on the randomization, double blinding and withdrawals or dropouts [30]. **The PEDro scale is a ten point questionnaire that scores studies from 0 to 10 based on the randomization, subject and assessor blinding, validity of outcome measures, appropriateness of treatment methods, proper statistical analysis, and withdrawals or dropouts management [31].
